# Addressing phase instability and charge recombination in pyrolysis-synthesized BiVO_4_ via DFT-guided Mo doping for enhanced performance

**DOI:** 10.1093/nsr/nwag148

**Published:** 2026-03-10

**Authors:** Sainan Zhang, Donghui Li, Nengcong Yang, Xueshang Xin, Jizhang Wang, Caidi Chen, Fuxiang Zhang

**Affiliations:** Department of Chemical Physics, University of Science and Technology of China, Hefei 230026, China; State Key Laboratory of Catalysis, Dalian Institute of Chemical Physics, Chinese Academy of Sciences, Dalian 116023, China; State Key Laboratory of Catalysis, Dalian Institute of Chemical Physics, Chinese Academy of Sciences, Dalian 116023, China; State Key Laboratory of Catalysis, Dalian Institute of Chemical Physics, Chinese Academy of Sciences, Dalian 116023, China; State Key Laboratory of Catalysis, Dalian Institute of Chemical Physics, Chinese Academy of Sciences, Dalian 116023, China; Center of Materials Science and Optoelectronics Engineering, University of Chinese Academy of Sciences, Beijing 100049, China; State Key Laboratory of Catalysis, Dalian Institute of Chemical Physics, Chinese Academy of Sciences, Dalian 116023, China; State Key Laboratory of Catalysis, Dalian Institute of Chemical Physics, Chinese Academy of Sciences, Dalian 116023, China; Center of Materials Science and Optoelectronics Engineering, University of Chinese Academy of Sciences, Beijing 100049, China; Department of Chemical Physics, University of Science and Technology of China, Hefei 230026, China; State Key Laboratory of Catalysis, Dalian Institute of Chemical Physics, Chinese Academy of Sciences, Dalian 116023, China; Center of Materials Science and Optoelectronics Engineering, University of Chinese Academy of Sciences, Beijing 100049, China

**Keywords:** density functional theory, metal doping, phase instability, solar-to-hydrogen, tandem device

## Abstract

Preparation of transparent bismuth vanadate (BiVO_4_, BVO) photoanodes via a scalable metal–organic decomposition (MOD) method has been long-term plagued by severe charge recombination and phase instability, preventing its application in solar-to-chemical energy conversion. Here, we employ density functional theory (DFT) calculations to screen dopants by evaluating their carrier transport properties and thermodynamic effects on phase stability. On this basis, Mo is identified and experimentally confirmed as a dual-role dopant that suppresses the formation of the inactive zircon-type tetragonal (z-t) while simultaneously enhancing charge-transport properties of BVO. Consequently, phase-pure monoclinic scheelite (m-s) Mo-doped BVO films prepared by the MOD method yield an optimized transparent photoanode (denoted 3Mo-BVO), exhibiting a remarkable charge separation efficiency of 96.2% at 1.23 V vs. reversible hydrogen electrode. Together with modification of the NiFeO_x_ co-catalyst, the 3Mo-BVO photoanode was used for assembly of a tandem device with a photovoltaic (PV) solar cell to achieve overall water splitting with a benchmarking solar-to-hydrogen (STH) efficiency of 4.7%.

## Teaser

Density functional theory-guided molybdenum doping stabilizes the desired monoclinic scheelite phase and enhances charge transport in bismuth vanadate, achieving record 4.7% solar-to-hydrogen efficiency.

## INTRODUCTION

The monoclinic scheelite (m-s) bismuth vanadate (BiVO_4_, BVO) has been extensively investigated as a photoanode owing to its excellent chemical stability, relatively narrow band gap (∼2.4 eV) and suitable band alignment for water oxidation [1,2]. However, its practical performance is constrained by poor bulk charge transport, stemming from low carrier mobility (0.044 cm^2^ V^−1^ s^−1^), short hole diffusion length (∼70 nm) [3,4], and critically, the formation of metastable zircon-type tetragonal (z-t) phase impurities often triggered by vanadium deficiency during synthesis. This undesired z-t phase possesses a wider bandgap (∼2.9 eV) and forms a type-I heterojunction with the m-s phase, thereby functioning as a charge recombination center that hinders carrier separation and drastically compromises photoelectrochemical (PEC) performance [5–7]. Consequently, suppressing phase impurities while enhancing bulk charge separation is essential for viable BVO films.

Currently, BVO thin films aretypically prepared by two-step electrodeposition (ED) or metal–organic decomposition (MOD). Although ED achieves high performance [5,8,9], its complexity and cost hinder large-scale fabrication [8,10,11]. In contrast, MOD offers a facile, scalable route to produce planar BVO thin films with high optical transparency, which is advantageous for tandem PEC integration [12]. Nevertheless, MOD-synthesized BVO suffers from persistent z-t phase impurities and limited charge separation efficiency. Extrinsic doping with elements like Mo [13,14], W [15] or P [16] has been extensively demonstrated to enhance carrier density and conductivity, leading to improved performance [17,18]. Moreover, theoretical studies have further elucidated how these dopants modify the electronic structure (e.g. band gap [19], effective mass [20]), and influence polaron migration [21,22]. However, most prior work suffers from two critical limitations that directly pertain to the formation of z-t phase impurities—one of the most detrimental factors limiting MOD-synthesized BVO. Firstly, these studies primarily focused on phase transitions between m-s and tetragonal scheelite (t-s) BVO [23], which offer only modest performance gains due to their structural similarity. Second, density functional theory (DFT) studies have largely neglected the critical issue of phase purity and failed to quantify the thermodynamic stability of the z-t phase relative to the m-s phase, particularly under pyrolytic synthesis conditions [24,25]. As a result, despite the crucial role of phase purity in governing the bulk charge transport in MOD-BVO films, theoretical phase-stability insights have rarely been integrated with experimental doping strategies.

Building on these considerations, we explored a theory-guided approach, utilizing DFT calculations to screen dopants by evaluating their thermodynamic effects on phase stability between m-s and z-t phases and carrier transport properties, thereby enabling simultaneous phase control and conductivity enhancement in one-step MOD synthesis. Among the candidates, Mo was identified as a promising dopant capable of favorably stabilizing the m-s phase while suppressing the formation of z-t phase through a mechanism distinct from and complementary to its conventional role in increasing conductivity. Guided by these predictions, we synthesized planar Mo-doped BVO films by the scalable MOD route and demonstrate that Mo doping exerts a dual function: fundamentally suppressing performance-limiting z-t phase impurities, a critical factor long overlooked in doping studies, while simultaneously enhancing carrier concentration to mitigate bulk recombination. The optimized 3Mo-BVO film displayed a charge separation efficiency (*η*_sep_) of 96.2% at 1.23 V vs. reversible hydrogen electrode (RHE). When integrated into a tandem configuration with a photovoltaic (PV) cell, the system delivered a record solar-to-hydrogen efficiency (STH) of 4.7% among reported planar BVO photoanodes. Our strategy of theory-guided dopant selection for concurrent conductivity enhancement and phase-purity control provides a generalizable framework for optimizing functional metal oxides.

## RESULTS AND DISCUSSION

### Theoretical calculations

**Figure 1.**
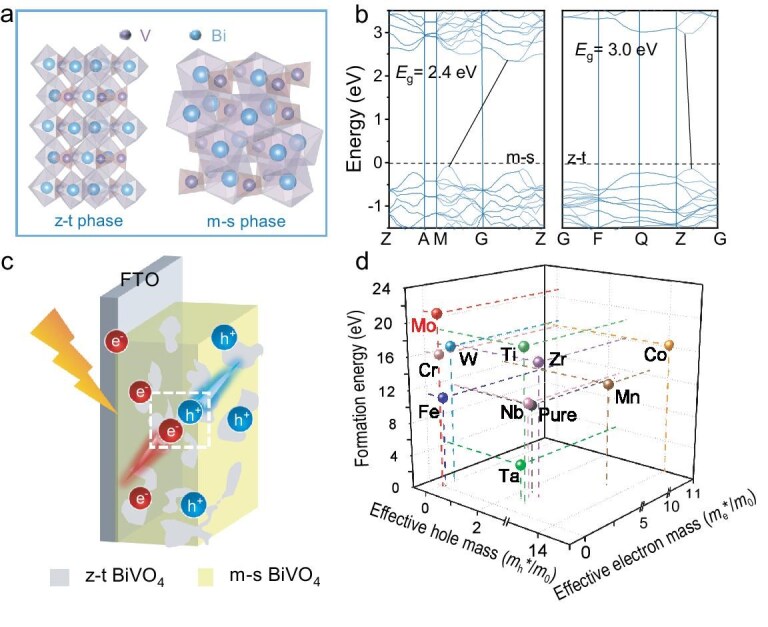
DFT calculations. (a) Crystal structure models of the zircon-type tetragonal (z-t) and monoclinic scheelite (m-s) phases BVO. (b) Energy band structure of m-s and z-t phases BVO; the Fermi level is set to zero. (c) Schematic illustration showing the charge separation in the m-s and z-t mixed-phases BVO photoanode. (d) Calculated monoclinic scheelite-zircon-type tetragonal phase-transition formation energy (Δ*E*_phase_) and effective masses of holes and electrons for different dopants.

In this study, we conducted systematic DFT calculations to explore the electronic and structural properties of m-s and z-t phases of BVO. Figure 1a displays the two crystal structure models of BVO employed in the DFT calculations. As shown in Fig. 1b and c, the band structure results reveal that the m-s and z-t phases form a type-I heterojunction upon contact, which facilitates bulk charge recombination and thereby hinders efficient charge carrier separation. Furthermore, the intrinsically large effective masses of both electrons and holes in the pristine m-s phase result in low carrier mobility, further limiting charge transport and separation efficiency [6].

To overcome these limitations, we examined the effect of doping with different metals on the carrier effective masses and the thermodynamic stability of the m-s and z-t phases of BVO. As summarized in Fig. 1d and Table S1, the normalized electron and hole effective masses (*m*_e_* and *m*_h_*) are plotted against the phase transition formation energy (Δ*E*_phase_) between the m-s and z-t polymorphs. Lower *m*_e_* and *m*_h_* values are indicative of higher carrier mobility and suppressed bulk recombination, whereas a higher Δ*E*_phase_ reflects greater thermodynamic stability of the m-s phase [20]. Notably, doping with Mo (*m*_e_* = 0.007, *m*_h_* = 0.031, Δ*E*_phase_ = 21.04 eV), Cr (*m*_e_* = 0.029, *m*_h_* = 0.034, Δ*E*_phase_ = 16.23 eV) and W (*m*_e_* = 0.397, *m*_h_* = 0.023, Δ*E*_phase_ = 16.89 eV) places these metals in the optimal zone near the mass-plane origin and high vertical coordinates, exhibiting minimized carrier masses and maximized Δ*E*_phase_. In contrast, Fe doping (*m*_e_* = 0.075, *m*_h_* = 0.076, Δ*E*_phase_ = 10.97 eV) offers outstanding transport properties yet negligible enhancement in phase stability relative to pristine BVO (*m*_e_* = 1.097, *m*_h_* = 2.235, Δ*E*_phase_ = 10.68 eV). Dopants like Co (*m*_e_* = 10.977, *m*_h_* = 14.034, Δ*E*_phase_ = 16.31 eV) is positioned at the far end of the mass plane, exhibiting prohibitively high effective masses that indicate sluggish carrier transport despite moderately favorable phase-transition energies. Although Mo, Cr and W all reside within the theoretical optimum, Mo uniquely combines a comparable electron effective mass and exceptionally high Δ*E*_phase_. This dual functionality—enhancing electron mobility and inhibiting the z-t phase transition—establishes Mo as the most promising dopant for experimental validation among all candidates examined. Based on this optimized doping metal, we then systematically investigated the effect of Mo doping concentration on the phase stability. A series of doping levels were evaluated, and it was found that the Δ*E*_phase_ increases sharply from 14.47 eV at 1% to over 21.04 eV at 3%, above which it plateaus (Fig. S1).

In addition, considering the differences in crystal structures of BVO, we further evaluated the effect of Mo, Cr and W doping with different metal cations. In the z-t phase of BVO, all four V–O bonds exhibit identical lengths (1.703 Å), while the m-s phase displays two distinct V–O bond lengths (1.769 and 1.693 Å) (Fig. S2) [26]. The resultant modifications in V–O and corresponding metal–oxygen (M–O) bond lengths are detailed in Table S2. Comparative calculations of dopants reveal that both Mo and W induce comparable local lattice distortions. Notably, the longer Mo–O bond (1.79 Å) raises the energy barrier for the phase transition, thereby favoring the stabilization of m-s phase. These structural insights further confirm Mo as the optimal dopant, introducing lattice distortions that destabilize the z-t phase configuration while promoting the m-s phase transition.

### Synthesis and characterizations

**Figure 2.**
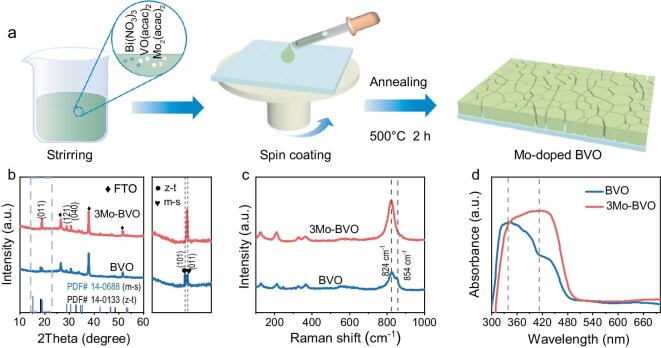
Synthesis and characterizations of Mo-doped BVO. (a) Schematic illustration of the synthesis of Mo-doping BVO. (b) XRD patterns, (c) Raman spectra, and (d) UV-vis absorption spectra of BVO and 3Mo-BVO photoanodes.

To experimentally validate this design paradigm, Mo-doped BVO (0–7 at%) was synthesized via the MOD method, as illustrated in Fig. 2a. Firstly, scanning electron microscopy (SEM) images of xMo-BVO showed negligible variations after doping (Figs S3 and S4), while elemental mapping of the optimized 3Mo-BVO confirmed homogeneous Mo distribution throughout the film, validated by linear scan consistency with Bi/V/O signals (Fig. S5) and individual elemental mapping (Fig. S6). The integration of Mo into the BVO lattice was further confirmed by the X-ray photoelectron spectroscopy (XPS) spectra (Fig. S7), where after addition of Mo atoms, Mo 3d XPS peaks appearing around 235.4 and 232.2 eV, and all of the core-level XPS peaks are shifted to higher binding energies with respect to the pristine BVO, indicating a modified local electronic environment around Bi and V. This observation is consistent with the substitution of V^5+^ by Mo^6+^, which slightly strengthens the overall metal–oxygen bonding [13,27]. Furthermore, the enhanced shoulder peak in the V 2p_3/2_ spectrum suggests a reduced V species (4+) was formed by increased electron density.

Building upon the confirmation of successful Mo doping, the crystal structure evolution across the Mo-doped series and specifically within the optimized 3Mo-BVO sample was systematically investigated using complementary techniques. X-ray diffraction (XRD) analysis (Fig. 2b, Fig. S8) reveals that a gradual weakening of the z-t-related features at 18.3° (JCPDS #14-0133) and a corresponding intensification of the m-s peaks can still be discerned with increasing Mo content. Upon 3% Mo doping, this z-t phase signature disappears, stabilizing into a pure m-s phase structure (JCPDS #14-0688). Raman spectra analysis (Fig. 2c, Fig. S9) provides molecular-level evidence for this phase evolution; the BVO sample exhibits two main peaks at 824 and 854 cm^−1^ corresponding to the symmetric V–O stretching vibrations of m-s and z-t phases, respectively [7,28]. Notably, the z-t phase-associated shoulder peak at 854 cm^−1^ shows progressive attenuation for 3Mo-BVO, accompanied by a slight low-energy shift of the m-s phase peak at 824 cm^−1^. This indicates elongation of the V–O bond [11,29] caused by Mo^6+^ replacing the smaller V^5+^ and introducing local lattice distortions. The ultraviolet-visible (UV-vis) spectra further corroborate these structural modifications (Fig. 2d, Fig. S10). The two distinct absorption peaks at the wavelength of 335 and 413 nm are clearly observed for undoped BVO, corresponding to the z-t and m-s phases, respectively, while increasing Mo doping gradually suppresses the z-t-associated absorption feature [6,30], in a manner consistent with the phase evolution observed in XRD patterns and Raman spectra. These results are consistent with the complete transition to the m-s phase, confirmed by corresponding DFT calculations and crystal structure analysis. Moreover, the calculated Δ*E*_phase_ for ≥3% Mo that induces complete phase transformation is ∼20 eV. By contrast, the Δ*E*_phase_ values for Cr (16.23 eV) and W (16.89 eV) are both below this critical threshold, providing a theoretical explanation for their inability to achieve complete phase stabilization.

To further semi-quantitatively assess phase purity, the relative content of the m-s and z-t phases in the BVO electrodes was estimated using UV-vis absorption spectroscopy. The synthesized phase-pure z-t reference [6] was first confirmed by XRD and UV-vis absorption spectra (Fig. S11). Quantitative analysis reveals a definitive trend: the m-s phase content increases from approximately 60% in undoped BVO to ∼97% in the 3Mo-BVO sample (Table S3). Crucially, apparent z-t phase characteristics in 3Mo-BVO spectra originate from inherent limitations of optical absorption spectroscopy for thin-film quantification, where the differences in surface roughness and film thickness collectively give rise to systematic errors. Taken together, these results demonstrate that Mo incorporation enables precise phase control through crystallochemical modification, thermodynamically favoring m-s phase formation and effectively suppressing metastable z-t phase nucleation.

### Photoelectrochemical performance and improved charge separation

**Figure 3.**
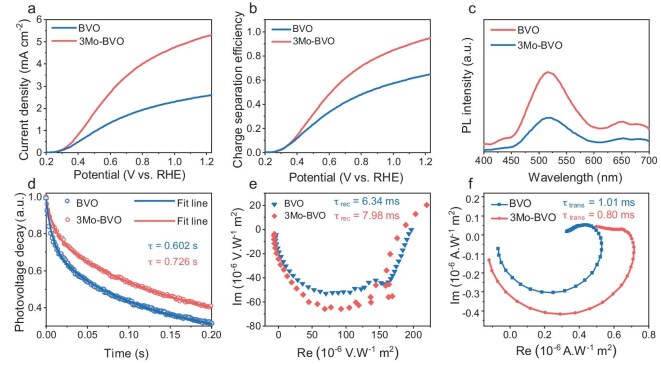
Photoelectrochemical performance of BVO and 3Mo-BVO. (a) Current density–voltage (*J*–*V*) curves. (b) Charge transport efficiency (*η*_sep_). (c) Steady-state photoluminescence (PL) spectra. (d) Transient photovoltage decay. (e) IMVS spectra measured at 0.6 V vs. RHE. (f) IMPS spectra measured at open-circuit for BVO and 3Mo-BVO photoanodes.

The PEC performance of Mo-doped and pristine BVO samples was preferentially evaluated in a 0.5 M KBi electrolyte containing Na_2_SO_3_ as a hole scavenger. As depicted in the current density–voltage (*J*–*V*) plots under AM 1.5G illumination (Fig. 3a), the pristine BVO exhibited a limited photocurrent density of 2.61 mA cm^−2^ at 1.23 V vs. RHE, while the optimized 3Mo-BVO photoanode achieved a substantially higher photocurrent density of 5.3 mA cm^−2^ at the same potential. Furthermore, the integrated photocurrent densities, according to Fig. S12, are 4.1 and 5.6 mA cm^−2^ for pristine BVO and 3Mo-BVO, respectively. The resultant *η*_sep_ values are increased dramatically from 65.4% in pristine BVO to 96.2% in 3Mo-BVO (Fig. 3b), further confirming the promotion effect of Mo doping on PEC performance. The optimization process is shown in Fig. S13; the doping concentration of 3% Mo yields the highest photocurrent density, which correlates with the optimal phase purity. This inverse relationship between performance and z-t phase content strongly suggests that these impurities act as major recombination centers. Notably, when doping exceeded 3%, the photocurrent density declined even though the structure remained purely m-s phase. This indicates that beyond a certain threshold, excessive Mo incorporation itself becomes detrimental [31].

To elucidate the mechanistic origin of the performance enhancement, the carrier dynamics and electronic properties were analyzed for different Mo-doping BVO samples. Electrochemical impedance spectroscopy (EIS) revealed a reduced semicircle radius for 3Mo-BVO compared to pristine BVO (Fig. S14a, Table S4), indicating lower charge transfer resistance (*R*_ct_) and enhanced interfacial charge transfer. Mott–Schottky (M–S) curves (Fig. S14b) confirmed that all samples display n-type characteristics with positive slopes, while 3Mo-BVO film exhibits the smallest slope corresponding to the highest charge carrier density among the series (Table S5), favorable for improving conductivity. However, for samples with >3% Mo doping, although the m-s phase-pure BVO is maintained, the increased slope in the M–S plots signals a decrease in donor density. This is attributed to a self-compensation mechanism wherein excessive Mo forms Mo–O–Mo bonds, which act as deep-level defects, reduce free electron concentration, and accelerate carrier recombination [27]. Crucially, although 7Mo-BVO possesses a near-identical donor density to undoped BVO (only 1.23% higher), it delivers a 28% photocurrent density increase under identical test conditions. This provides direct evidence that suppressing the z-t phase reduces recombination losses by eliminating z-t recombination centers, independently enhancing performance beyond doping effects.

Further advanced carrier dynamics characterization provided multiscale evidence for the performance enhancement of the optimized 3Mo-BVO compared to pristine BVO. Steady-state photoluminescence (PL) spectra (Fig. 3c) demonstrated recombination suppression in 3Mo-BVO, exhibiting significantly attenuated intensity at 510 nm. Transient photovoltage (TPV) measurements (Fig. 3d) revealed notably slower photovoltage decay in the optimized 3Mo-BVO (fitted lifetimes: 0.726 s) vs. BVO (0.602 s), indicating improved charge separation efficiency. Complementary insights from intensity-modulated voltage/photocurrent spectra (IMVS/IMPS) and corresponding Bode plot quantification (Fig. S15) revealed superior transport kinetics for 3Mo-BVO. This was evidenced by a prolonged electron lifetime (τ_rec_ = 7.98 ms) and a reduced charge transfer time (τ_trans_ = 0.80 ms), derived from Equations S5 and S6. Furthermore, the calculations based on Equations S7 and S8 and the film thickness (∼140 nm) indicated an increased carrier diffusion coefficient (*D*_n_ = 6.12 vs. 3.57 μm^2^ s^−1^) and extended diffusion length (*L*_n_ = 97.8 vs. 38.05 nm) for 3Mo-BVO compared to BVO (Fig. 3e and f, Table S6), confirming optimized bulk transport properties. The significantly reduced charge recombination and prolonged carrier lifetime are ascribed not merely to an incremental increase in carrier density, but more critically to the Mo-induced electronic structure modification. This key modification is directly correlated with the elimination of the z-t phase, as evidenced by our structural analysis. The phase purification establishes a robust framework for efficient charge transport and separation, which ultimately leads to the record performance. Supporting evidence comes from transient photocurrent decay (TPC) measurements (Fig. S16), which demonstrated significantly enhanced charge extraction for 3Mo-BVO, strongly suggesting that Mo-doping facilitates rapid electron transport and efficient charge collection at the fluorine-doped tin oxide (FTO) substrate. Additionally, open-circuit potential (OCP) measurements under dark and irradiated conditions revealed enhanced band bending in 3Mo-BVO (*V*_ph_ = 0.173 vs. 0.122 V), indicating a stronger built-in electric field at the semiconductor/electrolyte interface (Fig. S17). Collectively, this comprehensive analysis establishes that optimal Mo doping synergistically enhances PEC performance through dual mechanisms: electronic structure optimization via increased donor density, and, most importantly, crystal phase engineering via the stabilization of the monoclinic scheelite structure.

To experimentally verify Mo’s superiority over other candidates, Cr-doped BiVO_4_ (xCr-BVO, x = Cr at%) films were synthesized using the same procedure. UV-vis absorption spectra show that characteristic peaks of the residual z-t phase persist even with increasing Cr concentration (Fig. S18a). XRD analysis further confirms the presence of the z-t phase in 3Cr-BVO, similar to undoped BVO (Fig. S18b). Although Cr doping improves the carrier concentration reflected by lower *m*_h_*/*m*_0_ and *m*_e_*/*m*_0_, the photocurrent density remains only marginally higher than that of undoped BVO (2.6 vs. 2.3 mA cm^−2^ at 1.23 V vs. RHE; Fig. S19) and is significantly lower than that of 3Mo-BVO. This result underscores that complete phase stabilization is a critical prerequisite for achieving high PEC performance.

### Unassisted photoelectrochemical cell

**Figure 4.**
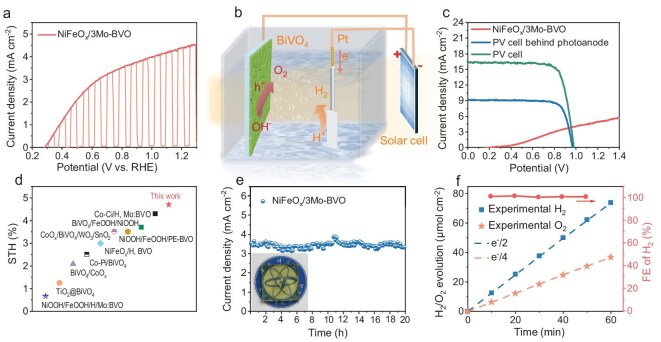
PEC performance of NiFeO_x_/3Mo-BVO. (a) *J*–*V* curve of NiFeO_x_/3Mo-BVO measured under chopped illumination. (b) Schematic illustration of the PV–PEC tandem device composed of a NiFeO_x_/3Mo-BVO photoanode and a GaAs PV cell. (c) Two-electrode *J*–*V* curves of NiFeO_x_/3Mo-BVO and GaAs PV cell with and without the photoanode filter. (d) STH comparison of MOD-synthesized BVO planar photoanodes. (e) Tandem device stability test under AM 1.5G illumination (optical image of 3Mo-BVO shown as inset). (f) H_2_ and O_2_ evolution and FE of NiFeO_x_/3Mo-BVO measured at 1.0 V vs. RHE under AM 1.5G illumination.

To further enhance the oxygen evolution reaction (OER) performance of the optimized 3Mo-BVO sample, NiFeO_x_ was introduced as a co-catalyst via a photo-assisted electrodeposition method. SEM characterization reveals a conformal coating on the surface (Fig. S20), and XPS analysis further verifies successful NiFeO_x_ loading through distinct Fe 2p and Ni 2p peaks (Fig. S21). The resulting NiFeO_x_/3Mo-BVO exhibits a lower overpotential and steeper water oxidation current slope than pristine BVO, indicating the excellent electrocatalytic activity of NiFeO_x_ for OER (Fig. S22). Correspondingly, the *J*–*V* curves (Fig. 4a) demonstrate a high photocurrent density of 4.53 mA cm^−2^ at 1.23 V vs. RHE under AM 1.5G illumination, with an outstanding applied bias photon-to-current efficiency (ABPE) value of 1.76%, achieved at a low applied potential of 0.63 V vs. RHE (Fig. S23). Given the excellent PEC properties and the planar structure with good light transmittance in Fig. S24, we further constructed a tandem PEC device for unassisted PEC water splitting by coupling the NiFeO_x_/3Mo-BVO photoanode with a single-junction GaAs PV cell, as depicted in the schematic diagram of Fig. 4b. The self-sustained system achieves a photocurrent density of 3.8 mA cm^−2^ at the maximum power point (Fig. 4c). Under these optimized conditions, the hybrid system achieves a STH of 4.7%, which is an excellent value among all the reported MOD-synthesized BVO planar photoanodes (Fig. 4d, Table S7) [32–41]. Importantly, extended durability testing confirms exceptional operational stability of the tandem device, with negligible performance degradation in Fig. 4e. Pre- and post-test XRD analyses verify complete retention of the m-s phase (Fig. S25). To decouple the contribution of the Mo-doped BVO film itself, we further examined NiFeO_x_/3Mo-BVO at 1.23 V vs. RHE under continuous operation (Fig. S26). Over 20 h, the photocurrent density decay remained below 3% with a similar planar structure confirmed by SEM (Fig. S27). Inductively coupled plasma mass spectrometry (ICP-MS) analysis (Table S8) reveals that both Mo and V remain below the detection limit, while small amounts of Ni and Fe are detected. This indicates that the slight photocurrent decay originates from minor dissolution of the NiFeO_x_ co-catalyst rather than the 3Mo-BVO lattice, thus demonstrating the intrinsic corrosion resistance of the Mo-doped film. Finally, hydrogen and oxygen evolution by PEC water splitting on NiFeO_x_/3Mo-BVO photoanodes at 1.0 V vs. RHE were measured by online gas chromatography. The amounts of H_2_ and O_2_ increased linearly to 73.95 and 36.74 μmol, respectively, after 1 h of operation (Fig. 4f). The average Faradaic efficiency (FE) approaches 100%, demonstrating excellent gas evolution capabilities of the NiFeO_x_/3Mo-BVO photoanode.

## CONCLUSIONS

In summary, we demonstrate the feasibility and effectiveness of a DFT-guided strategy for screening metal dopants in BVO with simultaneous consideration of carrier transport and phase stability. By explicitly evaluating both effective masses and the monoclinic scheelite–zircon-type tetragonal phase-transition formation energy, Mo was identified as a promising dual functionality dopant that can increase donor density while stabilizing the photoactive m-s phase against formation of the recombination-active zircon-type polymorph, which has long limited transparent BVO photoanodes prepared by pyrolysis routes. Guided by these insights, we fabricated highly efficient transparent planar 3Mo-BVO photoanodes that deliver a remarkable *η*_sep_ of 96.2% and an ABPE of 1.76%. When integrated in a PV–PEC tandem configuration, the system achieves an STH of 4.7%. We believe that this work illustrates how combining DFT analysis of transport and phase stability with targeted experiments can provide a general framework for rational dopant selection, and highlights the crucial, yet often overlooked, role of dopant-enabled phase purification beyond the conventional view of doping as merely a means to adjust electron transport.

## METHODS

### Synthesis of BiVO_4_ and Mo-doped BiVO_4_ samples

BiVO_4_ samples were fabricated following the typical procedure reported in our previous work [6]. Briefly, 0.6 M Bi(NO_3_)_3_·5H_2_O dissolved in dimethyl sulfoxide (DMSO) was mixed with stoichiometric VO(acac)_2_ (98%; Sigma–Aldrich), ultrasonicated for 30 min, and then filtered through a 0.22 μm polytetrafluoroethylene (PTFE) membrane. To prepare Mo-doped samples, MoO_2_(acac)_2_ (0–7 at%) was added. After depositing 50 μL of precursor onto FTO substrates (2 × 2 cm^2^) at a spin speed of 3500 r/min for 30 s, the resulting films were calcined at 500°C for 2 h with a heating rate of 5°C/min. The undoped reference is denoted BVO, while the Mo-doped series is denoted as xMo-BVO (x = Mo at%). Cr-doped BiVO_4_ (xCr-BVO, x = Cr at%) films were synthesized using the same procedure except that acetylacetonate molybdenum was replaced with acetylacetonate chromium.

### NiFeO_x_ co-catalyst deposition on the BiVO_4_ film

The NiFeO_x_ co-catalyst was deposited using a photo-assisted linear sweep voltammogram (LSV) method under AM 1.5G illumination. Briefly, 0.1 mol of H_3_BO_3_ was dissolved in 200 mL of deionized (DI) water and then KOH was added to give a 0.5 M borate buffer solution, with a pH of 8.3, then 20 mg of FeSO_4_·7H_2_O and 2 mg of NiSO_4_·6H_2_O were dissolved in the above solution before being washed with N_2_ for 20 min. LSV was performed at the potential of −0.3 to 0.5 V vs. Ag/AgCl under AM 1.5G illumination on the FTO side with a scan rate of 50 mV s^−1^ for 6–7 circles.

## Supplementary Material

nwag148_Supplemental_File
