# Supplementary material for: Addressing phase instability and charge recombination in pyrolysis-synthesized BiVO4 via DFT-guided Mo doping for enhanced performance
Source: Natl Sci Rev. 2026 Mar 10;13(10):nwag148. doi: 10.1093/nsr/nwag148 (PMC13228139; doi:10.1093/nsr/nwag148)
Supplement: nwag148_Supplemental_File [file nwag148_supplemental_file.pdf]

Addressing Phase Instability and Charge Recombination in Pyrolysis-Synthesized $BiVO_4$ via DFT-Guided Mo Doping for Enhanced Performance

Sainan Zhang†[1,2], Donghui Li†[2], Nengcong Yang[2], Xueshang Xin[2,3], Jizhang Wang[2], Caidi Chen[2,3], Fuxiang Zhang[1,2,3*]

[1]Department of Chemical Physics, University of Science and Technology of China, Hefei 230026, Anhui, China

[2]State Key Laboratory of Catalysis, Dalian Institute of Chemical Physics, Chinese Academy of Sciences, Dalian 116023, Liaoning, P. R. China

[3]Center of Materials Science and Optoelectronics Engineering, University of Chinese Academy of Sciences, Beijing 100049, China

[*]Corresponding author. Email: fxzhang@dicp.ac.cn

† These authors contributed equally to this work.

1. Experimental Section

1.1 Materials

FTO coated glass (TEC-15 14 Ω/sq, 2 cm × 2 cm), $Bi(NO_3)_3 \cdot 5H_2O$ (Sinopharm Chemical Reagent, AR), vanadium (IV)-oxy acetylacetonate (Alfa Aesar, 99.8%), citric acid (Sinopharm Chemical Reagent, AR), HNO_3 (Tianjin Kemiou Chemical Reagent, AR), DMSO (Sinopharm Chemical Reagent, AR), KOH (Sinopharm Chemical Reagent, AR), H_3BO_3 (Sigma-Aldrich, 99.5%), ethanol (Sinopharm Chemical Reagent, AR), $FeSO_4 \cdot 7H_2O$ (Sinopharm Chemical Reagent, AR), $NiSO_4 \cdot 6H_2O$ (Sinopharm Chemical Reagent, AR). and $MoO_2(acac)_2$ (98.0%; Sigma-Aldrich). The single-junction gallium arsenide photovoltaic cell was directly produced by Xia Yi Energy Company.

1.2 Synthesis of $BiVO_4$ and Mo-doped $BiVO_4$ samples

$BiVO_4$ samples were fabricated following the typical procedure reported in our previous work.[1] Briefly, 0.6 M $Bi(NO_3)_3 \cdot 5H_2O$ dissolved in DMSO was mixed with stoichiometric $VO(acac)_2$ (98%, Sigma-Aldrich), ultrasonicated for 30 min, and then filtered through a 0.22 μm PTFE membrane. To prepare Mo-doped samples, $MoO_2(acac)_2$ (0-7 at%) was added. After depositing 50 μL precursor onto FTO substrates (2×2 cm^2) at a spin speed of 3500 rpm for 30 s, the resulting films were calcined at 500 ℃ for 2 h with a heating rate of 5 ℃/min. The undoped reference is denoted BVO, while the Mo-doped series is denoted as xMo-BVO (x = Mo at%).

Cr-doped $BiVO_4$ (xCr-BVO, x = Cr at%) films were synthesized using the same procedure except that acetylacetonate molybdenum was replaced with acetylacetonate chromium.

1.3 $NiFeO_x$ cocatalyst deposition on the $BiVO_4$ film

The $NiFeO_x$ cocatalyst was deposited using a photo-assisted linear sweep voltammogram (LSV) method under AM 1.5G illumination.[1] Briefly, 0.1 mol H_3BO_3 is dissolved in 200 mL DI water and then KOH is added to give a 0.5 M borate buffer solution, with a pH of 8.3. Then, 20 mg of $FeSO_4 \cdot 7H_2O$ and 2 mg of $NiSO_4 \cdot 6H_2O$ were dissolved in the above solution before being washed with N_2 for 20 min. LSV is

performed at the potential of - 0.3 to 0.5V vs. Ag/AgCl under AM 1.5G illumination on the FTO side with a scan rate of 50 mV s^{-1} for 6–7 circles.

2. Characterization

The morphologies of samples were imaged by a scanning electron microscope FESEM (JSM-7900, JEOL), the elements of the samples were identified by the equipped energy dispersive X-ray spectroscopy (EDX). The TEM image and SAED pattern were obtained using a JEOL transmission electron microscope (JEM F200) at an accelerating voltage of 200 kV. The ultraviolet–visible absorption spectra (UV–vis) from 300 to 700 nm were taken on Shimadzu-UV2600 spectrophotometer. Powder X-Ray diffractions (XRD) were measured on a Rigaku SmartLab powder diffractometer operating at 40 kV and 200 mA with Cu Kα radiation (λ = 0.154 nm). Inductively coupled plasma atomic emission spectroscopy was conducted on Shimadzu ICPS-8100 to determine the metal content. The steady-state photoluminescence spectroscopy (PL) is FLS1000. XPS measurements were performed on a Thermo ESCALAB 250Xi with a monochromatized Al Kα source (15 kV, 10.8 mA). The calibration of binding energies obtained in the XPS spectral analysis was performed by referencing C 1s to 284.8 eV. The Raman instrument is Bruker Optics' Senterra spectrometer, which uses a 532 nm laser emitted by a solid-state laser as a light source.

3. (Photo)electrochemical measurements

All the photoelectrochemical (PEC) measurements were conducted in a typical three-electrode cell using the electrochemical workstation (Modulab XM, Solartron). A Xe arc lamp equipped with an AM 1.5G filter was used as the light source and the light intensity was calibrated to ca. 100 mW/cm^2 with a standard silicon cell detector (Newport). Current-voltage (J-V) curves under irradiation and dark were recorded with a scan rate of 20 mV·s^{-1}. The stability of the tandem device was tested under continuous AM 1.5G illumination. During testing, the electrolyte was periodically refreshed. Electrochemical impedance spectroscopy (EIS) Nyquist plots were collected with an AC voltage amplitude of 10 mV under AM 1.5G illumination (frequency range: 0.1 Hz~100 kHz). Photovoltage measurement was performed according to a previous report, open circuit potentials (OCP) of photoanodes were

measured in dark and under AM 1.5G illumination after stabilizing for enough time. The electrolyte solution was 0.5 M potassium borate electrolytes (KBi, pH = 9.5), 0.2 M Na_2SO_3 was added to the electrolyte as a hole scavenger. The counter and reference electrodes used were platinum foil and Ag/AgCl (3M KCl) electrode. The potential was converted to the RHE by the Nernst equation as below:[2]

$$E_{RHE} = E_{(Ag/AgCl)} + 0.059pH + E^{\theta}_{(Ag/AgCl)} \tag{S1}$$

where E_{RHE} refers to the converted potential *versus* RHE. The value of $E^{\theta}_{(Ag/AgCl)}$ is 0.197 V at ambient temperature (25°C) and $E_{(Ag/AgCl)}$ is the obtained potential versus Ag/AgCl.

Applied bias photon-to-current efficiency (ABPE) can be calculated using the following equation:[3]

$$ABPE\ (\%) = J \times (1.23 - V_{bias})/P_{total} \tag{S2}$$

Where J is the photocurrent density (mA cm^{-2}) obtained from the electrochemical workstation. V_{bias} refers to the applied bias versus RHE (V), and P_{total} is the total light intensity of AM 1.5G (100 mV cm^{-2}).

Mott-Schottky (M-S) curves were obtained in the voltage window of 0.5~1.2 V vs. RHE (increment: 10 mV, frequency: 1 kHz) with a 0.5 M borate buffer electrolyte (pH = 9.5) under dark. According to the M-S curves, the flat band potential can be obtained using the following equation:

$${C_{SC}}^{-2} = \frac{2}{e\varepsilon_0\varepsilon}(V - V_{fb}) \tag{S3}$$

$$N_d = \frac{2}{e\varepsilon\varepsilon_0}\left(\frac{d(1/C^2)}{dV}\right)^{-1} \tag{S4}$$

where C is the specific capacitance, the electronic charge (e) is 1.6 $\times 10^{-19}$ C, vacuum permittivity (ε_0) is 8.85 $\times 10^{-14}$ F cm^{-1}, and relative permittivity (ε) is 68 for $BiVO_4$, V is applied bias (vs. RHE). N_d is donor density for n-type semiconductor (cm^{-3}). The summarized flat potentials and N_d are listed in Table S5.

Control intensity modulated photocurrent/photovoltage spectroscopy (IMPS/IMVS) was obtained in Solartron Modulab XM to identify the several key processes involving charge transfer and surface recombination for PEC water oxidation in 0.5 M

KBi solution. IMPS was measured at 0.6 V versus RHE, while IMVS was measured at an open-circuit condition. The intensity-modulated light source was controlled by superimposing a sinusoidal modulation (10% amplitude) on DC illumination at different bias light intensities, and the frequency range was set from 100 kHz to 0.1 Hz. Electron lifetime (τ_{rec}) and transfer time (τ_{trans}) were calculated using equations 5 and 6:

$$\tau_{rec} = (2\pi\, f_{min}(IMVS))^{-1} \tag{S5}$$

$$\tau_{trans} = (2\pi\, f_{min}(IMPS))^{-1} \tag{S6}$$

Where f_{min} (IMPS) and f_{min} (IMVS) are the characteristic frequency at the lowest point of IMPS/IMVS Bode plots, respectively.

The carrier diffusion coefficient (D_n) and diffusion length (L_n) were calculated based on the formula:

$$D_n = d^2/(4\tau_{tran}) \tag{S7}$$

$$L_n = (D_n \times 4\tau_{rec})^{1/2} \tag{S8}$$

Where the film thickness (d) ≈110 nm and 140 nm of BVO and 3Mo-BVO. The calculated values are shown in Table S6. Under the given test conditions, the D_n and L_n of BVO are 3.57 $\mu m^2\ s^{-1}$ and 38.05 nm, while the values of 3Mo-BVO are 6.12 $\mu m^2\ s^{-1}$ and 97.8 nm, respectively.

4. Calculation

4.1 Calculation of theoretical photocurrent density

The light harvesting efficiency (η_{LHE}) of a semiconductor can be calculated using the following equation:[3]

$$\eta_{LHE} = 1 - 10^{-A} \tag{S9}$$

The light absorbance (A) is measured experimentally by UV-vis spectroscopy. According to the UV–vis light absorption curves of the BVO and 3Mo-BVO films shown in Figure 2d, the light harvesting efficiency (η_{LHE}) curves were obtained in Figure S7, their theoretical maximum absorbed photon-to-current density (J_{abs}) of BVO (4.10 mA cm^{-2}) and 3Mo-BVO (5.6 mA cm^{-2}) are obtained by integrating their product of the LHE with the solar photon flux, respectively.

Charge separation efficiency (η_{sep}), the yield of photogenerated holes that have

reached the semiconductor/electrolyte interfaces) can be calculated using the following equations:

$$\eta_{sep} = J_{Na2SO3}/J_{abs} \qquad \text{(S10)}$$

Where J_{abs} is the unity converted photocurrent density from the light absorption, while J_{Na2SO3} is the photocurrent density obtained in 0.5 M potassium borate electrolytes (pH = 9.5) with 0.2 M Na_2SO_3.

4.2 DFT Calculation

All Density Functional Theory (DFT) calculations were performed using the Vienna Ab initio Simulation Package (VASP) [4]. Exchange–correlation effects were treated with the Perdew–Burke–Ernzerhof (PBE) functional within the generalized gradient approximation (GGA) [5]. Electron–ion interactions were described by the projector augmented-wave (PAW) method [6], and van der Waals (vdW) interactions were accounted for via Grimme's DFT-D3 correction [7]. A plane-wave kinetic energy cutoff of 500 eV was consistently applied.

1. Band Structure and Effective Charge-carrier Mass Calculations

The electronic properties of pristine and doped bismuth vanadate ($BiVO_4$) in both monoclinic-scheelite (m-s) and zircon-type tetragonal (z-t) phases were investigated using a 2×1×2 supercell (96 atoms). For doped systems, one V atom was substituted by a dopant atom. Geometry optimizations were performed until the residual forces on all atoms were below 0.01 eV/Å. The Brillouin zone was sampled with a Γ-centered 4×4×4 k-point mesh, and the electronic self-consistency (SCF) was converged to 5×10^{-7} eV. To address the band-folding effect inherent in supercell calculations, the band unfolding technique, as implemented in the VASPKIT code [8], was employed to project the supercell wavefunctions onto the Brillouin zone of the corresponding primitive cell. Based on the unfolded band structures, the effective charge-carrier masses for electrons and holes were extracted using the Sumo code [9] by performing a least-squares parabolic fit to the energy-momentum dispersion at the band edges (VBM and CBM) along high-symmetry directions.

2. Phase Stability and Energetics Calculations

To evaluate defect formation energies and carrier transport properties while minimizing finite-size effects, a large (3×3×3) supercell containing 648 atoms was constructed. For the doped systems, the dopant concentration was set to approximately 3 at.% relative to the vanadium (V) atoms per supercell. Additionally, for molybdenum doping, a concentration gradient ranging from 1% to 7% relative to the V atoms was examined to assess concentration-dependent effects. Due to the high computational cost, a sequential optimization strategy was adopted: structures were first pre-relaxed using a Machine Learning Force Field (MLFF) [10] trained on ab initio data. The MLFF optimizations were converged until the total energy change was below 10^{-5} eV and forces were less than 0.05 eV/Å. Subsequently, high-precision DFT static calculations were performed on the MLFF-optimized geometries to obtain accurate total energies and charge densities, with an SCF convergence threshold of 10^{-5} eV. The Phase Transition formation energy was calculated using the following equation:

$$E_f = E_{\mathrm{tot}}[BVO, z-t] - E_{\mathrm{tot}}[BVO, m-s] \quad \text{(S11)}$$

where E_{tot} [*BVO*, *m-s*] and E_{tot} [*BVO*, *z-t*] represent the total energies of supercells comprising 3×3×3 unit cells, for both pure and doped phases of m-s and z-t phases BVO, respectively.

To maintain charge neutrality in doped systems, a valence-based compensation scheme was implemented by introducing intrinsic vacancies. For dopants with a higher valence state than V (supervalent), a Bi vacancy was introduced; for dopants with a lower valence (aliovalent), an O vacancy was created. This scheme was consistently applied across all calculations, including effective mass and phase stability evaluations, to ensure the physical validity of the charged defect states.

4.3 The Semi-quantitative Analysis by the UV-vis Absorption

The UV-vis absorption of the liquid phase is an effective method for chemical quantitative analysis, which is based on Lambert Beer's Law. For light absorption by solid thin films, the transmitted light intensity follows Equation.

$$A = \lg(1/T) = Kbc \tag{S12}$$

$$T(x) = \frac{I(x)}{I_0} = (1 - R)^2 \exp(-\alpha x) \tag{S13}$$

Where T, I, and R represent transmittance, light intensity, and reflectance; α is the light absorption coefficient, and x corresponds to light absorption depth. K is the molar absorption coefficient, b is the thickness of the light absorption layer, cc is the concentration or content.

According to the Lambert Beer's Law, for a series of solid films with the same preparation method, considering that they have similar thicknesses, it can be approximate that the value of b is same for them, so the relative content of phase can be calculated as below.

$$\frac{A_m}{A_t} = \frac{K_m b c_m}{K_t b c_t} \tag{S14}$$

$$\frac{c_m}{c_t} = \frac{K_t A_m}{K_m A_t} \tag{S15}$$

By combining equation S15 with the conservation of the total amount of phases (c_m + c_t =c_{total}), the relative content of the m-s phase f_m can be obtained:

$$f_m = \frac{c_m}{c_m + c_t} = \frac{1}{1 + \frac{c_t}{c_m}} = \frac{1}{1 + \left(\frac{K_t A_m}{K_m A_t}\right)^{-1}} \tag{16}$$

The A, K, and c represent the absorbance, molar absorption coefficient, and content, respectively. The value of $\frac{K_m}{K_t}$ can be obtained by the absorption ratio of the pure m-s phase (7Mo-BVO) and z-t phase (synthesized according to previous reports[1]), respectively. Based on this, the relative content of m-s phase in the electrode can be calculated by a semi-quantitative method, the relative content of m-s and z-t phases in BVO, Mo-BVO, 3Mo-BVO, 5Mo-BVO, and 7Mo-BVO, have shown in **Table S3.**

5. Discussion

Table S1. Comparative analysis of calculated monoclinic scheelite–zircon-type tetragonal phase-transition formation energy (ΔE_{phase}) and effective masses of holes and electrons for different dopants.

Dopants	ΔE_{phase}	Effect hole mass m_h^*/m_0	Effect electron mass m_e^*/m_0
Pure	10.67669	2.235	1.097
Mo	21.039	0.031	0.007
Cr	16.23	0.029	0.034
Ti	18.4639	2.61	0.5
Zr	16.18076	2.61	0.971
Nb	11.3775	2.395	0.822
Co	16.307	14.034	10.977
Fe	10.97	0.076	0.075
Mn	12.56	13.374	5.486
Ta	4.1328	2.315	0.642
W	16.89	0.023	0.397

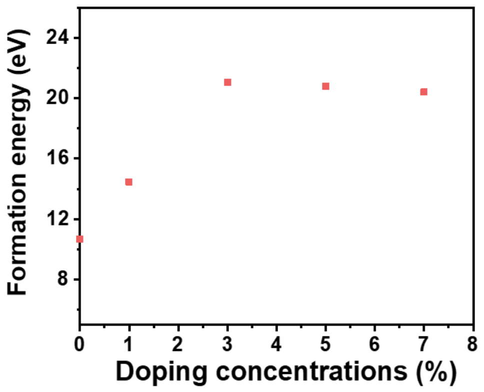

Figure S1. Comparative analysis of calculated monoclinic scheelite–zircon-type tetragonal phase-transition formation energy (ΔE_{phase}) for different Mo doping concentrations.

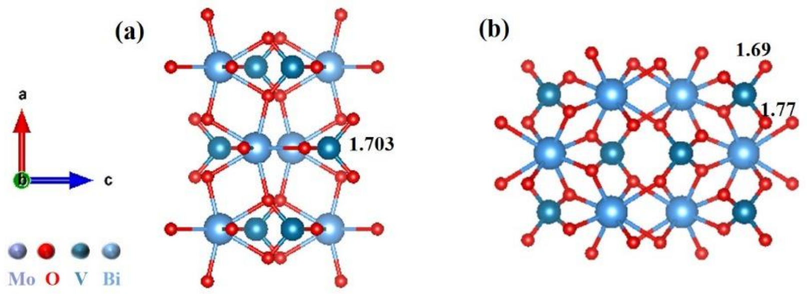

Figure S2. Crystal structures of (a) zircon-type tetragonal and (b) monoclinic scheelite BVO with bond lengths shown in Å.

Table S2. Comparative analysis of metal-oxygen (M-O) bonds and V-O for various

dopants in z-t BVO

Dopants	V-O (Å)	M-O bond (Å)
Mo	1.72~1.74	1.79
W	1.72~1.75	1.78
Cr	1.72~1.72	1.71

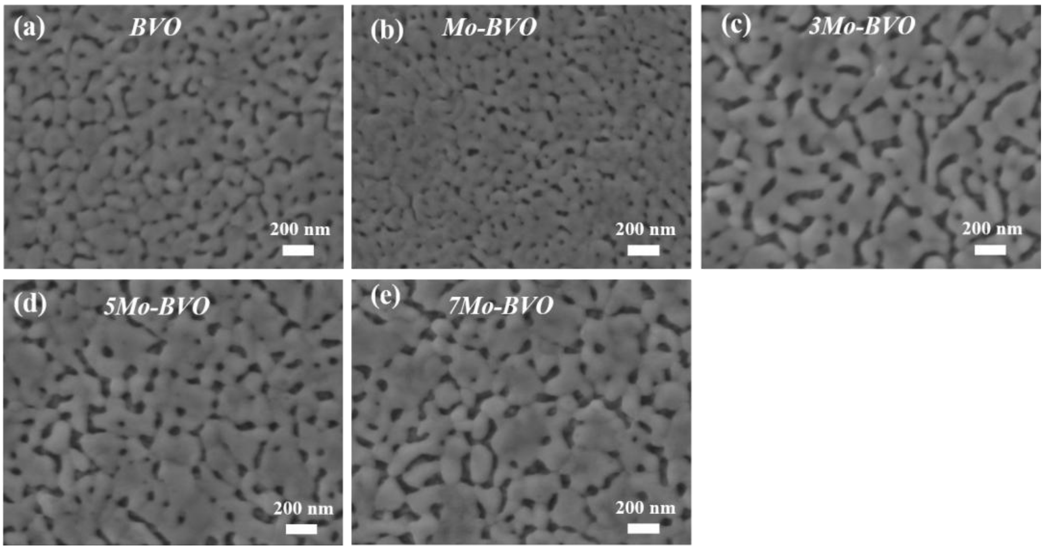

Figure S3. SEM images of (a) BVO, (b) Mo-BVO, (c) 3Mo-BVO, (d) 5Mo-BVO and (e) 7Mo-BVO.

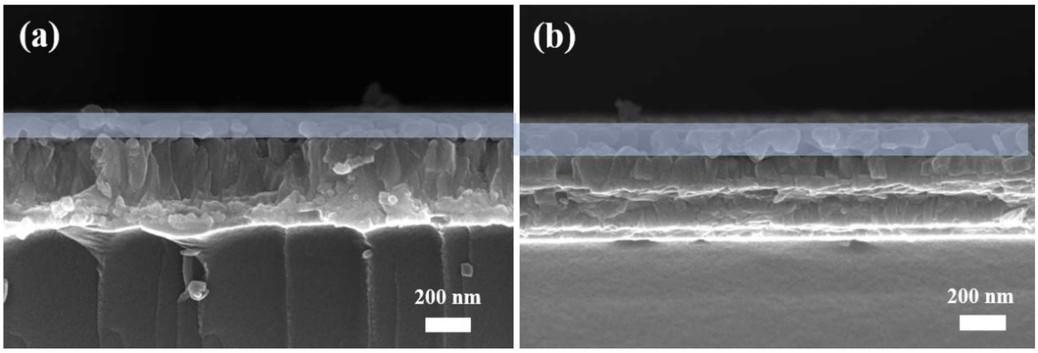

Figure S4. Cross section SEM images coated on FTO glass substrates of (a) BVO photoanode, (b) 3Mo-BVO photoanode.

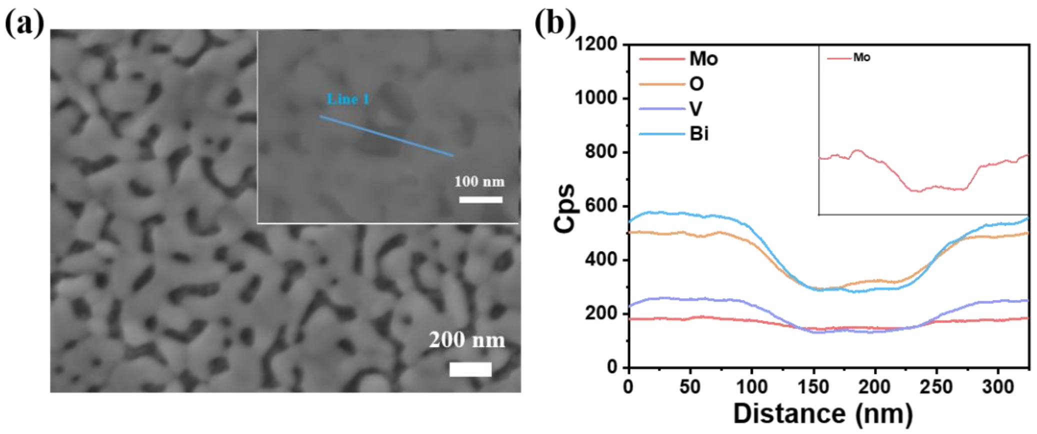

Figure S5. (a) SEM image of 3Mo-BVO photoanode and (b) the corresponding elemental distribution along line 1 illustration presented in (a).

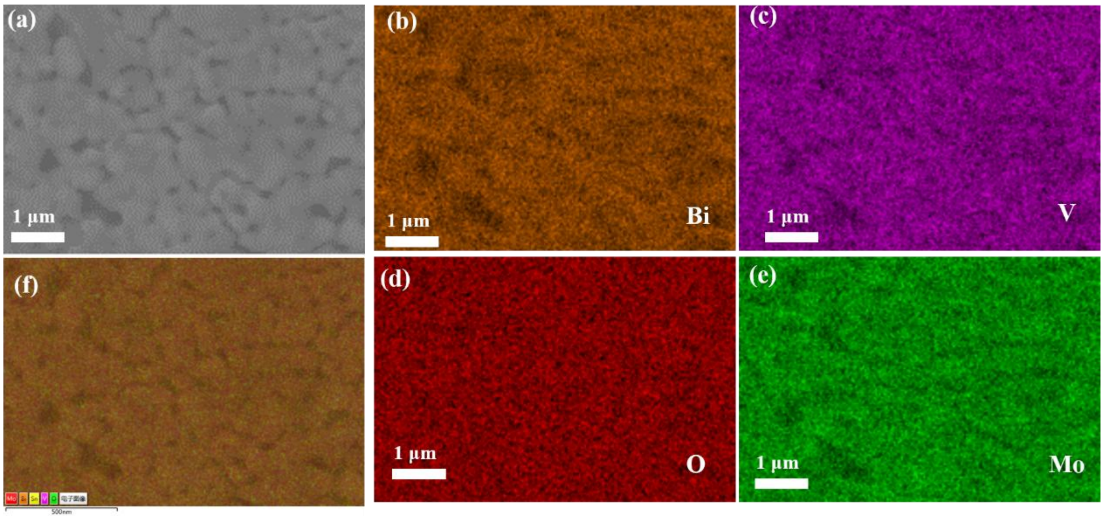

Figure S6. SEM-EDS mapping images of the 3Mo-BVO photoanode: (a) SEM, (b) Bi, (c) V, (d) O, (e) Mo and (f) All elements superimposition.

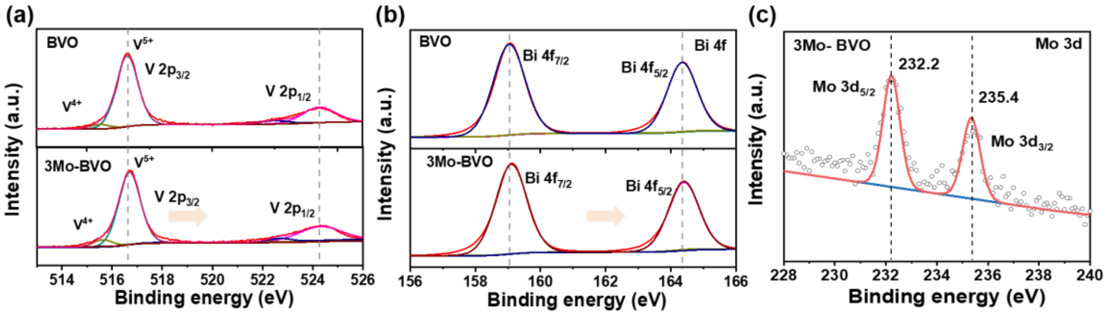

Figure S7. XPS spectra of BVO and 3Mo-BVO photoanodes: (a) Bi 4f, (b) V 2p, (c) Mo 3d.

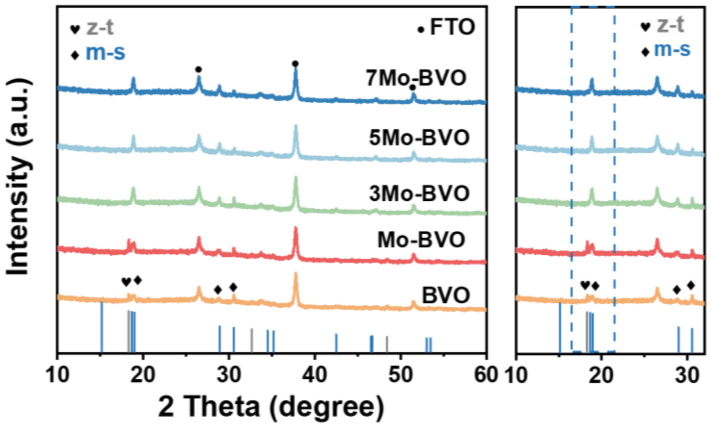

Figure S8. XRD patterns and local magnification of BVO and Mo-doped BVO samples.

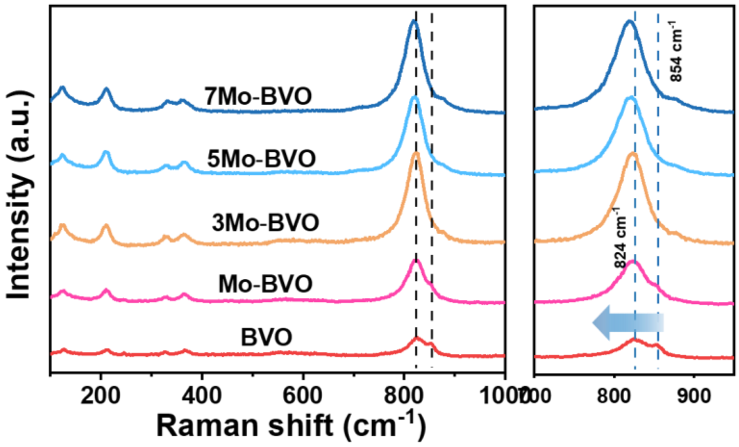

Figure S9. Raman spectra and local magnification of BVO and different Mo-doped BVO photoanodes.

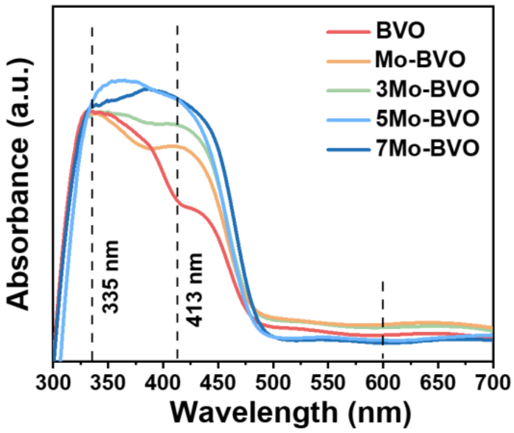

Figure S10. UV-vis absorption spectra of BVO and different Mo-doped BVO

photoanodes.

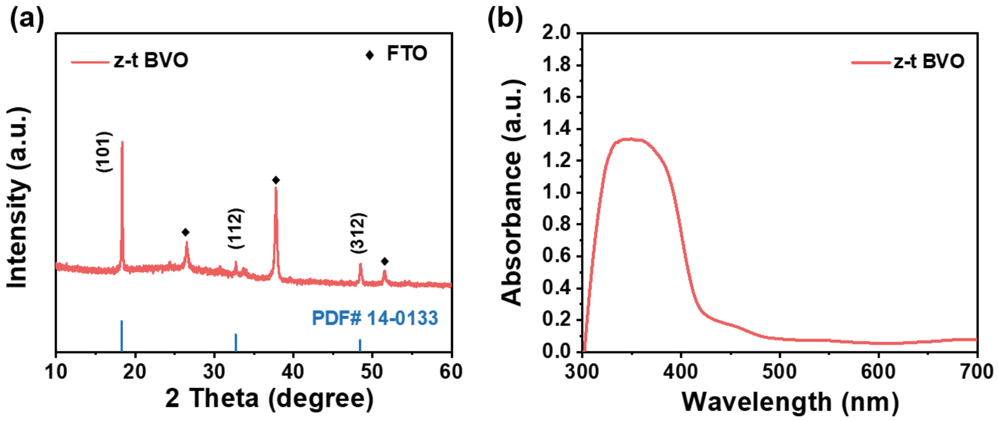

Figure S11. XRD pattern and (b) The corresponding UV-vis absorption spectroscopy of z-t phase BVO electrode.

Table S3. The percentage content of the m-s phase in BVO and Mo-doped BVO samples, calculated from UV–vis spectroscopy.

	BVO	Mo-BVO	3Mo-BVO	5Mo-BVO	7Mo-BVO
$Abs._{335nm}$	1.27	1.26	1.24	1.33	1.29
$Abs._{413nm}$	0.82	1.1	1.21	1.32	1.33
$Abs._{600nm}$	0.139	0.19	0.17	0.11	0.094
$Abs._{335nm}$- $Abs._{413nm}$	0.45	0.16	0.03	0.01	-0.04
m-s BVO	59.6%	85.0%	97.0%	99.2%	103.3%

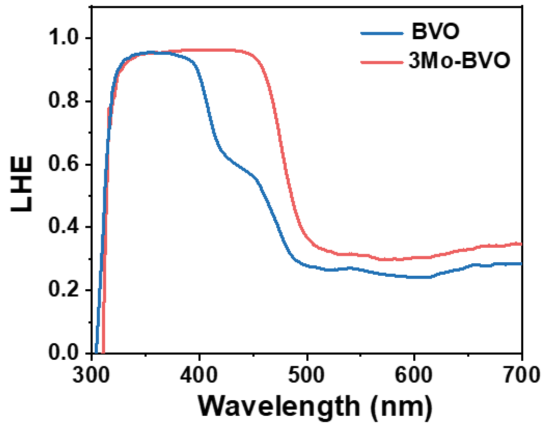

Figure S12. Light harvesting efficiency (LHE) of BVO and 3Mo-BVO photoanodes.

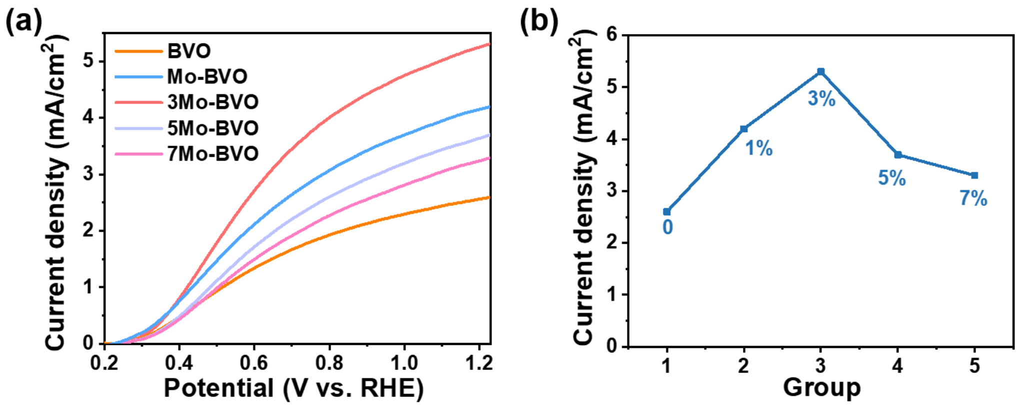

Figure S13. J-V curves of (a) BVO and xMo-BVO photoanodes tested in 0.5 M KBi containing 0.2 M Na_2SO_3 solution and (b) the corresponding line chart measured at 1.23 V vs. RHE.

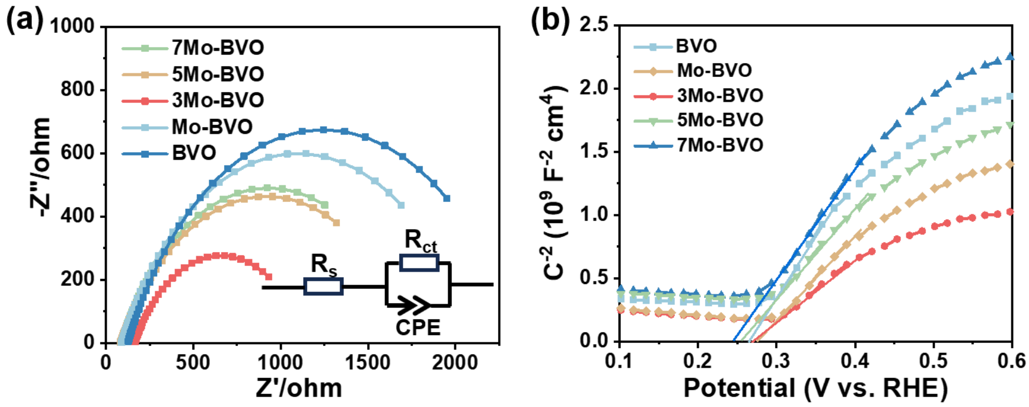

Figure S14. Charge transport dynamics of the BVO and xMo-BVO photoanodes: (a) EIS; (b) Mott-Schottky plots.

Table S4. The fitted results of EIS data using the equivalent circuit in Figure S14.

**Sample**	**R_{ct}**	**CPE-T**	**R_s**
BVO	2237	0.00020	123.7
Mo-BVO	2023	0.00024714	86.77
3Mo-BVO	991.8	0.00038992	162.5
5Mo-BVO	1679	0.00036098	80.91
7Mo-BVO	1698	0.00027823	82.56

Table S5. The calculated flat potential and donor density by M-S.

Sample	Nd (cm^{-3})
BVO	2.44×10^{20}
Mo-BVO	3.42×10^{20}
3Mo-BVO	4.39×10^{20}
5Mo-BVO	3.42×10^{20}
7Mo-BVO	2.47×10^{20}

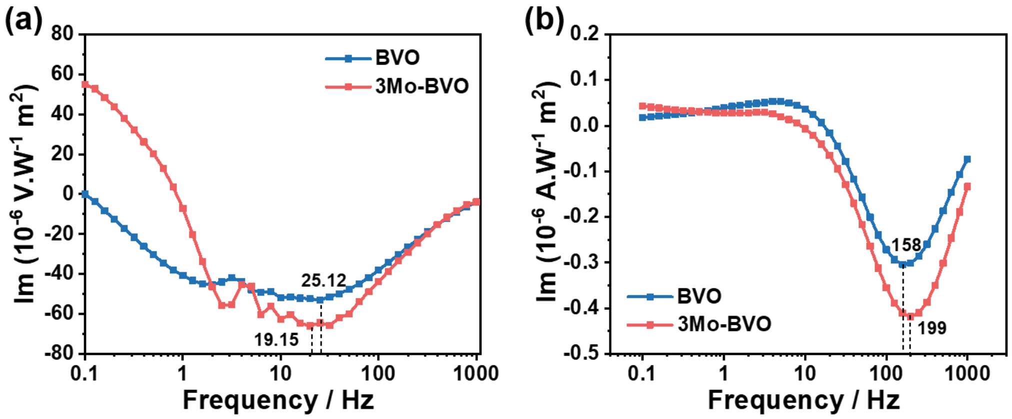

Figure S15. Bode plots of (a) IMVS and (b) IMPS spectra of BVO and 3Mo-BVO photoanodes.

Table S6. The calculated time constant of Electron lifetime (τ_{rec}) and transfer time (τ_{trans}).

	f_{min}/Hz	τ_{tran}/ms	f_{min}/Hz	τ_{rec}/ms	D_n(um^2/s)	(L_n)(nm)
BVO	158	1.01	25.12	6.34	3.57	38.05
3Mo-BVO	199	0.80	19.95	7.98	6.12	97.75

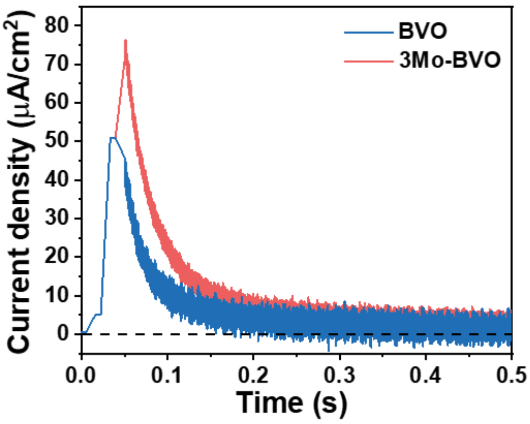

Figure S16. Transient photocurrent decay (TPC) of BVO and 3Mo-BVO

photoanodes.

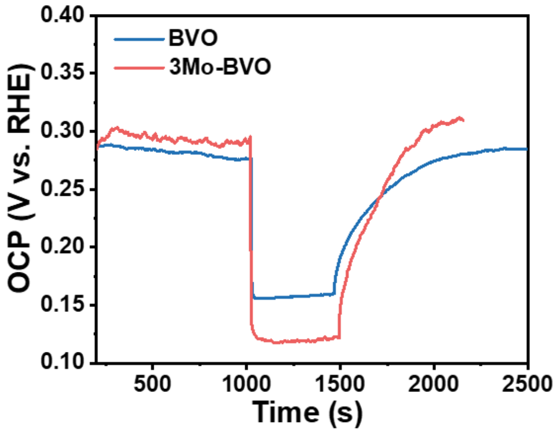

Figure S17. OCP plots under dark and light illumination of BVO and 3Mo-BVO photoanodes.

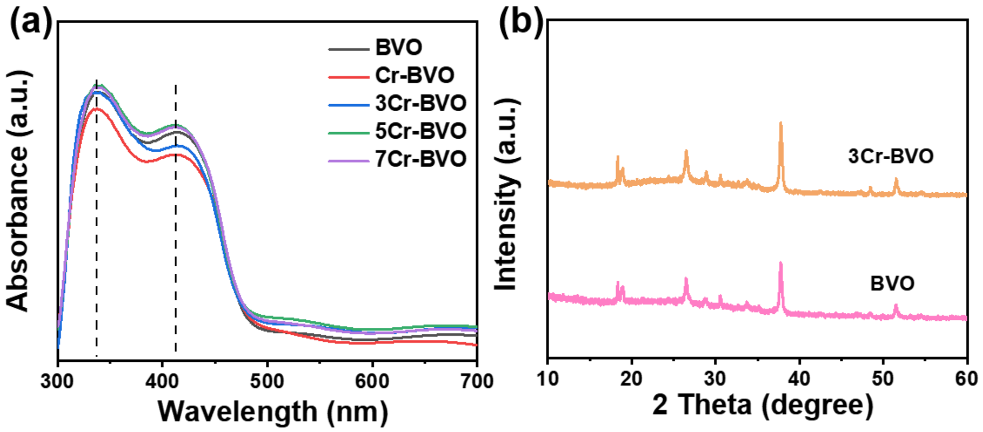

Figure S18 (a) UV-vis absorption spectra of different Cr-doping BVO samples, (b) XRD patterns of BVO and 3%Cr doping BVO samples.

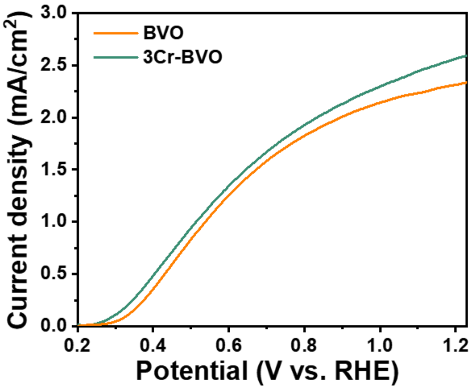

Figure S19 J-V curves of BVO and 3% Cr doping BVO samples.

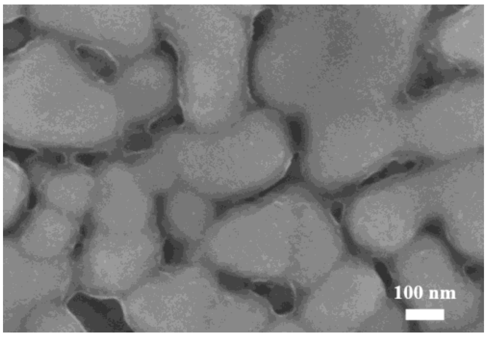

Figure S20. SEM image of $NiFeO_x$/3Mo-BVO photoanodes.

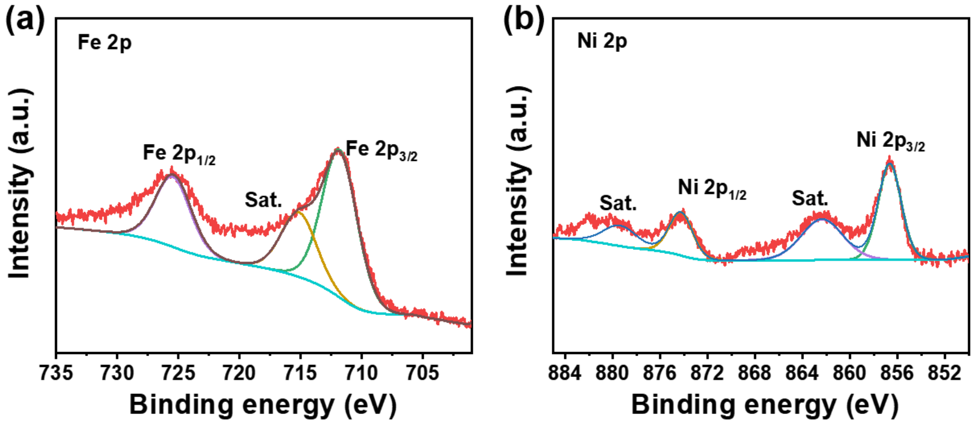

Figure S21. XPS spectrum of $NiFeO_x$/3Mo-BVO photoanodes. (a) Fe 2p, (b) Ni 2p

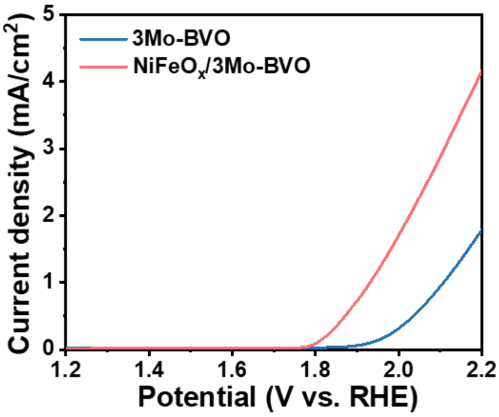

Figure S22. J-V curve of 3Mo-BVO and $NiFeO_x$/3Mo-BVO photoanodes in dark.

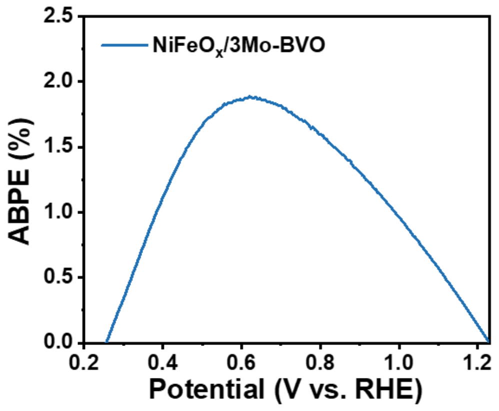

Figure S23. ABPE of NiFeO$_x$/3Mo-BVO photoanode.

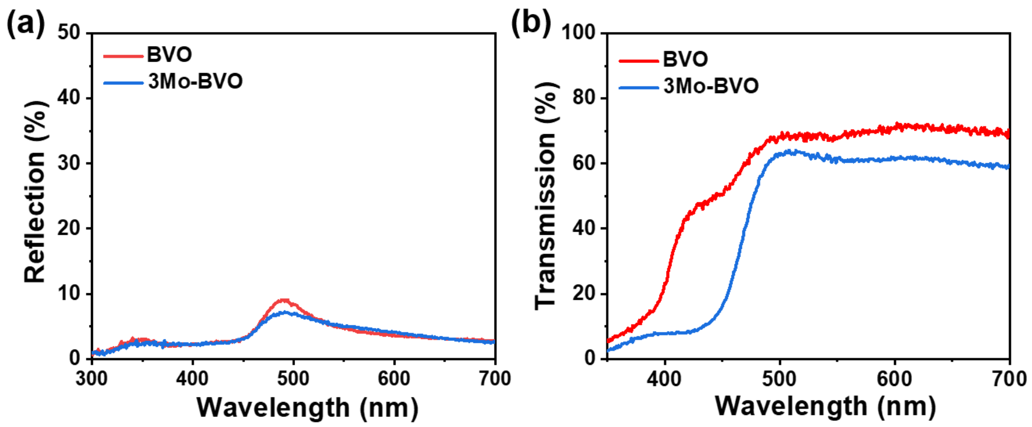

Figure S24. (a) Reflection and (b) Transmittance spectra of BVO and 3Mo-BVO photoanodes.

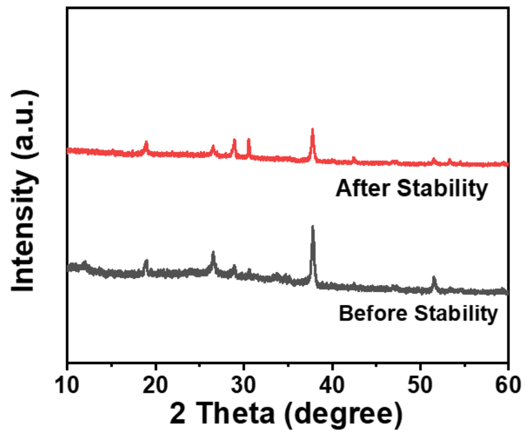

Figure S25. XRD pattern of NiFeO$_x$/3Mo-BVO after stability test.

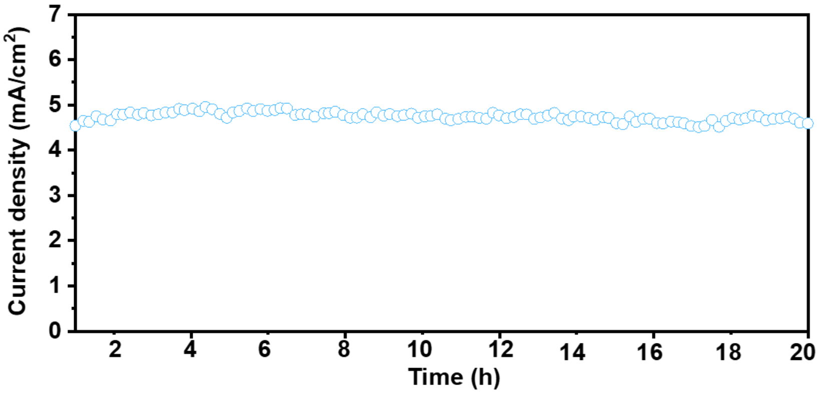

Figure S26. Stability test of $NiFeO_x$/3Mo-BVO at 1.23 V vs. RHE under AM 1.5G illumination.

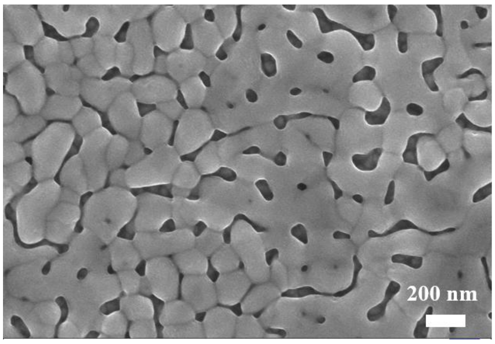

Figure S27. SEM image of $NiFeO_x$/3Mo-BVO after stability test.

Table S7. MOD-fabricated BVO-based planar photoanodes in tandem devices for unbiased water splitting.

Photoanode	In tandem with	Current density (mA cm^{-2})	STH	Ref.
Co-Ci/H, Mo:$BiVO_4$	PSC	4.8	4.3%	[11]
TiO_2@$BiVO_4$	PSC	1.3	1.24%	[12]
Co-Pi/$BiVO_4$	PSC	2.2	2.5%	[13]
CoO_x/$BiVO_4$/WO_3/SnO_2	PSC	3.1	3.5%	[14]
$BiVO_4$/CoO_x	pn^+ Si/TiO_2/Pt Si SHJ	3.3	2.09%	[15]
$BiVO_4$/FeOOH/NiOOH	Si	--	3.7%	[16]
FeOOH/NiOOH/PE-BVO	Si	3.0	3.5%	[17]
NiOOH/FeOOH/Mo:$BiVO_4$	Pt/CdS/$CuGa_3Se_5$ /(Ag,Cu)$GaSe_2$	3.4	0.67%	[18]
$NiFeO_x$/H,BVO	Cu_2O/Ga_2O_3/TiO_2/RuO_x	--	3%	[19]
$NiFeO_x$/3Mo-BVO	GaAs	4.53	4.7%	This work

PE = Photoetching

PSC = Perovskite solar cell

Table S8 ICP-MS analysis of the 0.5 M KBi electrolyte (pH = 9.5) after 20 h continuous operation of $NiFeO_x$/3Mo-BVO photoanode.

Sample	Fe (μg/mL)	Ni (μg/mL)	Mo (μg/mL)	V (μg/mL)
$NiFeO_x$/3Mo-BVO	0.00768	0.003519	0	0

Reference

1. Yang N; Zhang S; Xiao Y; Qi Y; Bao Y; Xu P; Jin S; Zhang F, Insight into the Key Restriction of $BiVO_4$ Photoanodes Prepared by Pyrolysis Method for Scalable Preparation. *Angew. Chem. Int. Ed.* 2023, **62**: e202308729.

2. Chen Z; Jaramillo T F; Deutsch T G; Kleiman-Shwarsctein A; Forman A J; Gaillard N; Garland R; Takanabe K; Heske C; Sunkara M; McFarland E W; Domen K; Miller E L; Turner J A; Dinh H N, Accelerating materials development for photoelectrochemical hydrogen production: Standards for methods, definitions, and reporting protocols. *J. Mater. Res.* 2010, **25**: 3-16.

3. Liu B; Wang X; Zhang Y; Xu L; Wang T; Xiao X; Wang S; Wang L; Huang W, A $BiVO_4$ Photoanode with a VO_x Layer Bearing Oxygen Vacancies Offers Improved Charge Transfer and Oxygen Evolution Kinetics in Photoelectrochemical Water Splitting. *Angew. Chem. Int. Ed.* 2023, **62**: e202217346.

4. G. Kresse av J F, Efficiency of ab-initio total energy calculations for metals and semiconductors using a plane-wave basis set. *Comput. Mater. Sci.* 1996, **6**: 15-50.

5. Perdew J P; Ernzerhof M; Burke K, Rationale for mixing exact exchange with density functional approximations. *J. Chem. Phys.* 1996, **105**: 9982-9985.

6. John P. Perdew K B, Matthias Ernzerhof, Generalized Gradient Approximation Made Simple. *Phys. Rev. Lett.* 1996, **77**: 3865–3868.

7. Joubert D, From ultrasoft pseudopotentials to the projector augmented-wave method. *Phys. Rev. B* 1999, **59**: 1758–1775.

8 V. Wang, N. Xu, J.C. Liu, G. Tang, W.T. Geng, VASPKIT: A User-Friendly Interface Facilitating High-Throughput Computing and Analysis Using VASP Code, *Comput. Phys. Commun.* 2021, **267**: 108033.

9 Ganose, A. M., Jackson, A. J. & Scanlon, D. O. sumo: Command-line tools for plotting and analysis of periodic ab initio calculations. *J. Open Source Softw.* 2018, **28:** 717.

10. Wu Z; Zhou L; Hou P; Liu Y; Wang R; Guo T; Liu J-C, A Machine Learning Interatomic Potential Data Set and Model for Catalysis with Local Fine-Tuning to Chemical Accuracy. *JACS Au* 2025, **5:** 6151–6161.

11. Jin Hyun Kim Y J, Ju Hun Kim, Ji Wook Jang, Hyun Jun Kang, Young Hye Lee, Dong Suk Kim, Yongseok Jun,; and Jae Sung Lee, Wireless Solar Water Splitting Device with Robust Cobalt-Catalyzed, Dual-Doped $BiVO_4$ Photoanode and Perovskite Solar Cell in Tandem: A Dual Absorber Artificial Leaf. *ACS nano* 2015, **9**: 11820–11829.

12. Zhang X; Zhang B; Cao K; Brillet J; Chen J; Wang M; Shen Y, A perovskite solar cell-TiO_2@$BiVO_4$ photoelectrochemical system for direct solar water splitting. *J. Mater. Chem. A* 2015, **3**: 21630-21636.

13. Chen Y S; Manser J S; Kamat P V, All solution-processed lead halide perovskite-

$BiVO_4$ tandem assembly for photolytic solar fuels production. *J. Am. Chem. Soc.* 2015, **137**: 974-81.

14. Baek J H; Kim B J; Han G S; Hwang S W; Kim D R; Cho I S; Jung H S, $BiVO_4/WO_3/SnO_2$ Double-Heterojunction Photoanode with Enhanced Charge Separation and Visible-Transparency for Bias-Free Solar Water-Splitting with a Perovskite Solar Cell. *ACS Appl. Mater. Interfaces* 2017, **9**: 1479-1487.

15. He Li B L, Shijia Feng, Huimin Li, Tuo Wang, Jinlong Gong, Construction of uniform buried pn junctions on pyramid Si photocathodes using a facile and safe spin-on method for photoelectrochemical water splitting. *J. Mater. Chem. A* 2020, **8**: 224–230.

16. Liu B; Wang S; Feng S; Li H; Yang L; Wang T; Gong J, Double-Side Si Photoelectrode Enabled by Chemical Passivation for Photoelectrochemical Hydrogen and Oxygen Evolution Reactions. *Adv. Funct. Mater.* 2020, **31**: 2416474.

17. Feng S; Wang T; Liu B; Hu C; Li L; Zhao Z J; Gong J, Enriched Surface Oxygen Vacancies of Photoanodes by Photoetching with Enhanced Charge Separation. *Angew. Chem. Int. Ed.* 2020, **59**: 2044-2048.

18. Kim J H; Kaneko H; Minegishi T; Kubota J; Domen K; Lee J S, Overall Photoelectrochemical Water Splitting using Tandem Cell under Simulated Sunlight. *ChemSusChem* 2015, **9**: 61-66.

19. Pan L; Kim J H; Mayer M T; Son M-K; Ummadisingu A; Lee J S; Hagfeldt A; Luo J; Grätzel M, Boosting the performance of Cu_2O photocathodes for unassisted solar water splitting devices. *Nat. Catal.* 2018, **1**: 412-420.
